# Effect of Supplements on Endurance Exercise in the Older Population: Systematic Review

**DOI:** 10.3390/ijerph17145224

**Published:** 2020-07-20

**Authors:** Alejandro Martínez-Rodríguez, Bernardo J. Cuestas-Calero, María Hernández-García, María Martíez-Olcina, Manuel Vicente-Martínez, Jacobo Á. Rubio-Arias

**Affiliations:** 1 Department of Analytical Chemistry, Nutrition and Food Science, Faculty of Sciences, University of Alicante, 03690 Alicante, Spain; amartinezrodriguez@ua.es; 2 Alicante Institute for Health and Biomedical Research (ISABIAL Foundation), 03010 Alicante, Spain; 3 Faculty of Sport, San Antonio Catholic University of Murcia, 30107 Murcia, Spain; bjcuestas@alu.ucam.edu; 4 Faculty of Health Sciences, University of Alicante; 03690 Alicante, Spain; mhg30@alu.ua.es (M.H.-G.); mmo36@alu.ua.es (M.M.-O.); 5 Faculty of Health Sciences, Miguel de Cervantes European University, 47012 Valladolid, Spain; mvmartinez11006@alumnos.uemc.es; 6 LFE Research Group, Department of Health and Human Performance, Faculty of Physical Activity and Sport Science-INEF, Universidad Politécnica de Madrid, 28040 Madrid, Spain

**Keywords:** diet, nutritional supplements, physical activity, older adults

## Abstract

Background: Ageing is associated with changes of physical and physiological parameters, but there is evidence that regular physical activity could minimize these effects. Additionally, the older population presents a great risk of suboptimal nutrition. Therefore, the purpose of this study was to review the evidence of nutritional strategies and endurance exercises in the older population. Methods: A systematic review was performed based on the preferred reporting items for systematic reviews and meta-analysis (PRISMA) statement. The search was carried out in three different databases: PubMed, Web of Science, and SPORTDiscus. Results: Eight studies were included in the present review. The use of caffeine and beta-alanine supplementation with proteins have been found to be beneficial in both sexes. In older women, a balanced diet, an increase in protein, supplementation with beta hydroxy methyl butyrate, and supplementation with sodium bicarbonate have been favorable. However, no benefit has been seen in older men with sodium bicarbonate or ubiquinone supplementation. Nevertheless, the use of supplements should be prescribed according to individual characteristics and physical activity. Conclusions: Caffeine and high protein supplement with beta-alanine may provide positive effects in the older population. In addition, in older women, bicarbonate supplementation and beta-hydroxyethyl butyrate (HMB), lysine, and arginine supplementation have shown positive effects on exercise performance.

## 1. Introduction

Worldwide, the older population (over 60) is growing faster than any other group, due to a longer life expectancy and a lower birth rate [1]. However, the ageing of the population can be considered a success of public health policies and socio-economic development. Ageing is also a challenge for today’s society, which must adapt and improve the functional capacity of the older population, their participation in the society, and their individual security [2].

Ageing has been represented as a sequence of physical and physiological changes, specifically on muscle mass, known as sarcopenia. This involves a progressive loss of skeletal muscle mass, associated mainly with an age increment that determines a loss of strength, with the risk of generating a disability, decreasing quality of life, and even ending in death. This reflects a reduction in anabolism and an increase in catabolism, together with a reduced capacity for muscle regeneration [3,4,5,6]. Because of the decrease in strength and mobility, independence is reduced, which increases the risk of falls, with the consequent increase in the incidence of fractures and impairment of recovery, thereby increasing the morbidity and demand for health and social care [7,8].

It is often difficult to distinguish between biological ageing and physical inactivity, because physical inactivity can cause further biological deterioration, leading to accelerated ageing. With ageing, there is a natural decay in physiological function, and this is complicated because, in turn, society becomes more sedentary over the years [9]. As a result of physical inactivity and age, muscle strength decreases, and this muscle weakness can compromise sports performance and daily living activities [3]. It is true that the biological process of ageing cannot be stopped with physical activity, but there is evidence that regular physical activity can minimize the physiological effects that occur with a sedentary lifestyle. In addition, there are also indications that regular physical activity reduces the risk of developing chronic diseases and has improvements on both physiological and cognitive levels in older people, such as slowing or delaying cognitive decline and increasing aerobic capacity, muscle strength, muscle mass, and bone density, so all healthy older people should perform regular physical activity and avoid an inactive lifestyle [10]. Additionally, a meta-analysis performed by Chou et al. (2012) [11], which investigated the effect that exercise had on physical function, activities of daily living, and quality of life in the elderly, gave evidence that exercise is beneficial for increasing speed of walking, improving balance, and improving performance in the activities of everyday life of older people. However, the findings suggest that there are no significant differences in the quality of life between frail elderly who are trained and untrained.

Exercise practices have been studied to determine if they reduce the risk of falls in the older population. Results showed how balance training may be effective. Furthermore, stretching and flexibility training have also been studied, and there is some evidence that flexibility can be increased in the main joints. However, it is not established how much or what type of range of motion exercises are the most effective [10]. Strength training by itself is of particular value to older people, because it improves muscle strength and muscle mass [5,6,10]. Nevertheless, the most common type of physical activity among older people is endurance exercises. Dallosso et al. (2003) [12] evaluated the daily activities carried out by 507 people between 65 and 74 years old and 537 people of 75 years or more. Most of the exercises that they performed involved maintenance of the house and the garden, as well as leisure activities, such as swimming and cycling, but by far the most practiced activity was walking. Comparing by sex, women are less physically active than men, in terms of aerobic exercise, and have less strength in the lower body [13,14].

Body composition measurements have shown that fat-free body mass has been positively associated with physical activity and negatively with age [15]. A higher level of physical activity is associated with greater muscle mass. A longitudinal evaluation of 3 years in men and women over 65 years showed that physical leisure time activity did not prevent the loss of muscle mass; however, a higher level of physical activity was associated with greater muscle mass [16]. Another longitudinal study also found that in older women a greater amount of protein was associated with a greater fat-free mass five years later [17]. However, muscle mass does not necessarily increase after training, but improvements can be observed in individual muscles, such as the vastus lateral, and intermediately after an exercise without changes in total lean mass [18]. But the question remains whether an active lifestyle can delay unwanted changes in body composition, such as decreased lean mass.

Regarding nutritional strategies, the older population is one of the groups that presents the greatest risks of nutritional problems due to ageing, as well as psychological, social, and economic changes, so the best strategy to address those issues is prevention and early detection [19]. Adequate nutritional support improves the quality of life, thereby reducing the degree of functional dependence of the elderly [20].

In situations where diseases or injuries occur, good availability of amino acids is important, since, in the absence of nutrients, the muscle is the main source of nutrients for protein synthesis, which causes a reduction in muscle volume [21,22]. The usefulness of supplements has been highlighted due to the action on the skeletal muscular system, which, together with physical activity (mainly endurance), can reduce the impact of osteoporosis, sarcopenia, and ageing in general [15]. In addition, it has been observed that a nutritional intervention in combination with vitamin and/or protein supplements improves quality of life and muscle strength, decreases depressive symptoms, and facilitates activities of daily living [23,24]. At present, to the best knowledge of the authors, there are no bibliographic reviews regarding nutritional strategies and supplements in older people who perform endurance exercises. However, such reviews do exist on strength exercises, since these types of exercises most benefit muscle mass. Therefore, the aim of this study was to review and study the effects of nutritional strategies on body composition and/or performance in the older population engaged in endurance exercise.

## 2. Materials and Methods.

### 2.1. Data Sources and Searches

This systematic review was carried out in accordance with the guidelines of preferred reporting items for systematic reviews and meta-analysis (PRISMA) [25,26].

The source of data collection was direct consultation and access, using the Internet for the scientific literature library of the PubMed, Web of Science, and SPORTDiscus databases. Searches in the databases were performed independently by two authors (A.M.-R and B.J.C.-C.).

For documentary recovery, the keywords “older person” were used: “OLDER PEOPLE” or “OLDER ADULTS” or “ELDERLY” or “AGED”. The Boolean operator “AND” was used to combine these descriptors with “ENDURANCE” and “NUTRITION” or “DIET”. The search equations can be reproduced at any time in the corresponding databases. A search was conducted for each possible combination of the above keywords in each database used. The date of the last update of the search was October 2019.

### 2.2. Inclusion and Exclusion Criteria

The final choice of documents was made according to the fulfilment of the inclusion and exclusion criteria described below. The inclusion criteria were the following: a) randomized clinical trials; b) studies in the English or Spanish language; c) older population; and d) carried out with endurance sports or publications whose subjects are trained in endurance sports. The review also included articles in which an intervention was carried out with supplementation and for those dealing with specific nutrition in older people who perform sports or physical endurance activity. Studies were excluded if they were reported in a) books or book chapters; b) dissemination articles; and c) patents. In addition, publications whose study population was not elderly and those that lacked inclusion of endurance sport were excluded.

### 2.3. Study Selection and Data Extraction

Two reviewers (A.M.-R. and B.J.C.-C.) independently extracted data from the included studies. Data collection took place by extracting information from each study included in this review. The data extracted were characteristics of the subjects, intervention period, measures taken to obtain the results, results, conclusions, type of exercise, and body composition of the subjects.

## 3. Results

### 3.1. General Characteristics of Studies

**Figure 1:**
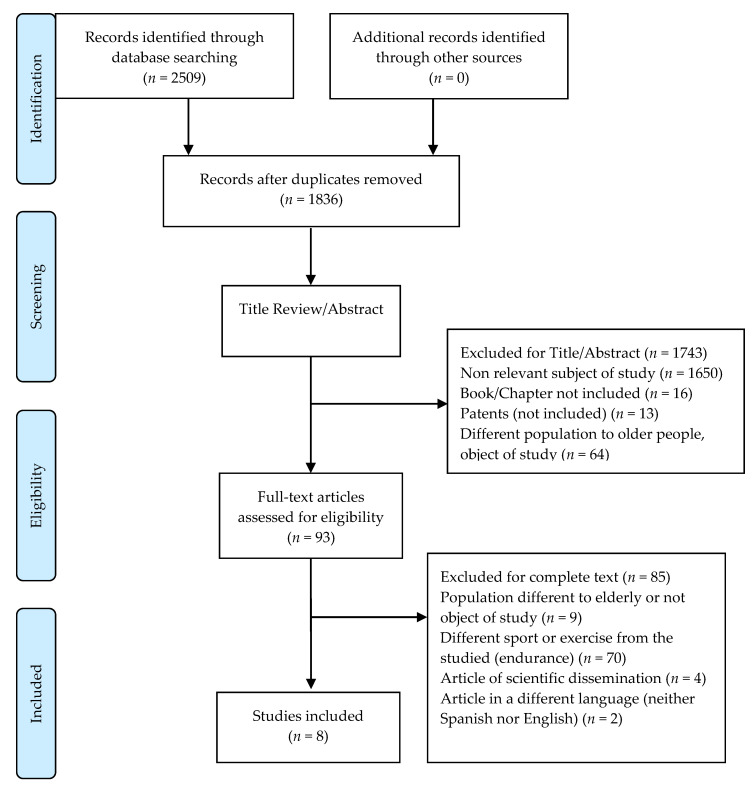
Preferred reporting items for systematic reviews and meta-analysis (PRISMA) flow diagram.

A total of eight studies were included in this review. The search in the PubMed, Web of Science, and SPORTDiscus databases resulted in a total of 2509 references (734 in PubMed, 1611 in Web of Science, and 164 in SPORTDiscus), of which 1836 were not duplicated. After removing the duplicate articles and analyzing the title and abstract, 1743 articles did not fit the subject under study (Figure 1). Finally, 93 articles were evaluated as full texts. Of these, 85 were excluded. Therefore, a total of eight studies were included in this review [27,28,29,30,31,32,33,34]. Seven of these studies were published between 2004 and 2014, although there was one from 1995. All were conducted in developed countries (three in the USA, one in the UK, two in Finland, one in Denmark, and one in Belgium) and had sample sizes in the range of 19–113 participants [27,28,29,30,31,32,33,34].

### 3.2. Risk-of-Bias Assessment

The methodological quality of the studies was assessed with the PEDro scale. Risk of bias was assessed independently by two authors (A.M.-R. and B.J.C.-C.). The purpose of the PEDro scale is to help quickly identify which of the randomized clinical trials may have sufficient internal validity (criteria 2–9) and sufficient statistical information to make their results interpretable (criteria 10–11). An additional criterion (criterion 1) relates to external validity [35]. Table 1 shows the results of the methodological quality of the studies measured by the PEDro scale.

The first item of the PEDro scale was not considered in this review, as it relates to the assessment of the external validity of the studies. Therefore, only items 2–11 were selected for the analysis of methodological quality. Consequently, the maximum score of an article will not be higher than 10 points and the minimum score may be 0 points.

The PEDro scale score ratings have not been validated, but it is highly desirable that six of the studies have a score between 8 and 9 out of 10 and that four studies have a score between 6 and 7 out of 10. This means that all the studies analyzed in this review were of moderate to high quality, with scores greater than or equal to 6/10 on the PEDro scale, therefore having strong internal validity.

### 3.3. Intervention

Table 2 shows the characteristics of the participants and the intervention of each study. Of the studies examined, four were conducted on both men and women [27,28,31,32], three conducted their intervention only on women [29,30,33], and one study was conducted only on men [34].

Regarding studies that examined both women and men, some carried out a training intervention, but others carried out specific endurance testing interventions. In terms of nutritional and supplementation strategies, each study carried out different interventions. Two of the studies [27,32] carried out the intervention with caffeine, although with different doses, and both obtained beneficial results for the participants. McCormack et al. (2013) [28], using a high protein nutritional supplement and two different amounts of beta-alanine, obtained good results to be taken into account. Dawson-Hughes et al. (2010) [31], studied muscle endurance as a “isokinetic muscle endurance of knee extensors and flexors, which were assessed on a Cybex II isokinetic dynamometer; where, after a period of warm-up and familiarization, subjects performed 25 maximal contractions at 240°/s”. In addition, they performed an intervention with sodium bicarbonate, potassium, or sodium chloride and they obtained positive results in women, but not in men. No significant results in terms of body composition were found in any of the studies.

As for the studies carried out only on women, all except the study by Flakoll et al. (2004) [33] carried out specific training and all were different from each other. The nutritional strategies followed in these studies were also disparate: Verschueren et al. (2011) [29] performed a vitamin D intervention, but no beneficial results were obtained. In the study by Sillanpaa et al. (2010) [30], the subjects followed the Finnish nutritional recommendations with 47 ± 6% of carbohydrates, 19 ± 3% of protein, and 32 ± 4% of fat, with no benefit. Finally, Flakoll et al. [33] performed an intervention with a mixture of specific nutrients—beta-hydroxyethyl methyl butyrate (HMB), lysine, and arginine—and obtained beneficial results in older women. Some studies found significant changes in body composition [30,33], with increases in muscle mass in one study and increases in fat-free mass in another.

In the study by Laaksonen et al. (1995) [34], the only study carried out only on men, no specific training was performed; all were trained subjects, but an ergometer endurance test was performed, which compares the intervention with ubiquinone in older and younger men, but no advantageous results were obtained in the older men. There were also no significant changes in the body composition of the participants in this study.

In both sexes, caffeine supplementation and supplementation with a high protein supplement and beta-alanine are beneficial for older people who engage in endurance exercise. Sodium bicarbonate, potassium, or sodium chloride have benefits only in women, reducing nitrogen excretion and reducing the loss of performance and muscle mass, as does HMB, lysine, and arginine supplementation, improving muscle function, strength, and protein synthesis. Supplementation with Vitamin D, following Finnish nutritional guidelines, and supplementation with ubiquinone did not have an endurance-increasing effect on the older population.

## 4. Discussion

The aim of this review was to present, while making comparisons between the sexes, the physical and physiological changes experienced by older people as a result of endurance interventions and nutritional supplements. It has been observed that more studies have been conducted in older women than in older men, and the results have shown that there are different nutritional strategies that, along with endurance training, are beneficial for this population. Ageing is associated with an increase in adiposity in sedentary individuals that increases the prevalence of obesity and the comorbidities associated with obesity [36,37]. A sedentary lifestyle is also associated with the loss of muscle mass and strength, which increases the risk of falls and bone fractures with age [38,39,40]. Therefore, identifying sports and nutritional strategies can help older adults promote body mass and fat loss while maintaining muscle mass and strength to help reduce the risk of age-related comorbidities and/or injuries and to improve health markers and functional capacity.

Nutritional requirements in the elderly are difficult to determine due to physiological changes, which can affect the nutritional status. Daily protein needs are increased in the elderly for many reasons, such as resistance to anabolism, lower postprandial availability of amino acids, sarcopenia, and disease-related protein catabolism [41]. Despite the differences that older people have in terms of general health and physiological status, the recommended daily amounts of protein are the same for adults of all ages (0.8 g protein/kg body mass/day) [41]. However, evidence suggests recommendations for optimal protein intake for adults over 65, which are between 1 and 1.2 g/kg of body mass/day [42,43,44,45,46].

In addition, suboptimal nutrition and resistance to anabolic stimuli have been related to skeletal muscle loss in old people. Nevertheless, adequate ingestion of basic nutrients, like amino acids, could contribute to muscle protein metabolism during an endurance training period [47].

Therefore, the supply of nutrients after exercise is important for the maintenance of skeletal muscle (proteins and carbohydrates). Sillanpaa et al. (2010) observed that a balanced diet, together with endurance training, increases muscle power in older women [30]. However, the nutritional needs of trained and untrained older people will be different [48] and the use of specific supplements will also depend on individual characteristics, including age, physical condition, the sex of the person, sports practice, or activity carried out at a specific time and the objective the person seeks with the intake of the supplement [27,31,33,49]. When it comes to hydration, although water requirements are no different in younger and older adults, older people are more likely to have inadequate water intake. Dehydration and electrolyte imbalance are common, since the sensation of thirst decreases with age, as well as the efficiency of renal and pulmonary mechanisms. Therefore, fluid intake should be increased, and it is recommended that older people consume 1.2–2 L of water per day to maintain the fluid balance and consume water before, during, and after exercise to prevent dehydration [50].

In reference to supplements, various types have been found in the literature that can potentially improve the performance or health status of older people who perform sports or physical endurance activity. Each supplement has its own characteristics and objectives of use and will be classified as stimulants, HMB, buffers, or antioxidants.

Caffeine has been well recognized for its exciting and anti-depressive properties, which produce a stimulation of the mood and antisoporifers, which reduce fatigue and increase physical performance capacity [3,27,51]. Caffeine also increases the conversion of reserve lipids into free fatty acids and can thus be used as a source of energy and save the use of muscle glycogen stores [49].

Duncan et al. (2014) [27] demonstrated that an acute intake of caffeine (3 mg·kg^−1^ of body mass) improves functional performance and manual dexterity in older men and women, so it can improve the ability to perform daily tasks. Norager et al. (2005) [32] also found that a caffeine intake of 6 mg/kg body mass increases cycling endurance, resistance to isometric flexion of the arm, and perceived exertion in older people. Both studies measured the effect 60 min after caffeine intake, as the maximum concentration of caffeine in blood plasma is 1 h after intake. These findings provide evidence of the beneficial effect of caffeine in older people. Information on the recommended dose of caffeine for the elderly varies between a dose of 200 mg and relative doses from 1.5 mg·kg^−1^ of body mass to 9 mg·kg^−1^ of body mass [52,53]. However, a dose in the range of 3–6 mg·kg^−1^ of body mass is considered optimal to achieve physical and mental effects and to minimize the adverse effects of caffeine [54].

It is important to take into account the possible adverse effects of caffeine; therefore, it should be known that it can interact with other supplements and nutrients, such as sodium bicarbonate, creatine, and carbohydrates. Although there is no clear evidence on the safe maximum amounts of caffeine, specialists recommend not to exceed 500 mg/day of caffeine [36].

HMB is a compound derived from leucine that influences muscle protein catabolism, cell membrane integrity, and sarcolemma stabilization [51]. It is attributed to an anticatabolic action, since it reduces the degradation of proteins and cell damage that occur during intense exercise. It has been proposed that the anti-catabolic effects often observed with leucine supplementation during periods of stress are mediated by HMB [36].

It has also been seen that supplementation with HMB (1.5 to 3 g/day) reduces markers of muscle catabolism and increases lean mass and strength in sedentary subjects at the start of the training period [49,51]. In the study by Flakoll et al. (2004) [33], with an intake of 2 g of HMB, 5 g of arginine, and 1.5 g of lysine HCl for 12 weeks, the functionality, strength, fat-free mass, and protein synthesis in older women improved. Therefore, supplementation with HMB positively affects muscle health in older women.

Beta-alanine is a precursor of carnosine, the main function of which is to act as an intramuscular hydrogenation buffer. The decrease in muscle carnosine levels can lead to a decrease in muscle buffering capacity, decreasing the ability to eliminate hydrogen ions during anaerobic activities [28].

McCormack et al. (2013) [28] showed how beta-alanine supplementation and a high protein ingestion can improve physical work capacity, muscle quality, and function in older men and women, but no difference was shown between a supplementation of 800 mg and 1200 mg of beta-alanine. In addition, there were no significant changes in body mass, lean soft tissue, or fat mass. In this study, the controversy remains that the effect found could not be attributed to beta-alanine supplementation, if not to the high protein ingestion through protein supplements.

Sodium bicarbonate is an alkalizing substance that acts as a buffer in an acid medium [51]. Metabolic acidosis promotes protein degradation and nitrogen excretion, and Dawson-Hughes et al. (2010) [31] found that a maintained sodium bicarbonate administration decreases nitrogen excretion and improves performance in healthy postmenopausal women, but not in men.

Positive effects of sodium bicarbonate in older men may not have been found due to the relationship of the dose with the body size. In relation to sodium bicarbonate supplementation, it has also been seen that chronic or repeated use of sodium bicarbonate supplements before interval training sessions can improve adaptation to the training, as well as improve performance during [36].

Regarding the role of vitamins, Verschueren et al. (2011) [29] studied the changes that occurred in older women after use of a vitamin D supplement once a day, comparing the effects of a high dosage (1600 IU) and a conventional dosage (880 IU). The two studied groups, also undergoing vibration platform training (EPV), had no significant differences. Strength and muscle mass did not change significantly, and a higher dose of vitamin D did not show muscle benefits in dynamic muscle strength, hip bone mineral density (BMD), or serum vitamin D levels compared to conventional doses in older women. Campbell and Geik (2004) [55] conducted a review, in which they found no evidence that supplementation with any specific micronutrient (vitamin or mineral) can improve performance (as long as there is no specific deficiency).

Coenzyme Q10, also known as ubiquinone, is a fat-soluble benzoquinone, a nonessential nutrient found primarily in animal foods and, in low amounts, in vegetables. In the human organism, it is mainly located in the heart and skeletal muscle. It acts in the production of ATP in the electronic transport chain and has an important antioxidant effect by inhibiting lipid and protein peroxidation and eliminating free radicals [51]. However, in the study conducted by Laaksonen et al. (1995) [34], no ergogenic effects were observed in trained older men. After supplementation with 120 mg of ubiquinone for 6 weeks, ubiquinone was ineffective in reducing fatigue and improving aerobic and anaerobic function in older men.

Therefore, it has been observed that caffeine improves performance, manual dexterity, and performance of day-to-day tasks and increases endurance in both men and older women [27,32]. Likewise, beta-alanine supplementation, together with a protein supplement, can improve physical work capacity, muscle quality, and function in both sexes [28].

In older women, it has been seen that a balanced diet, along with endurance training, increases muscle power [30]. As for more favorable nutritional strategies in women, it has been seen that supplementation with HMB, arginine, and lysine can improve functionality, strength, fat-free mass, and protein synthesis [33]. Moreover, sodium bicarbonate supplementation can decrease nitrogen excretion and improve performance [31]. However, supplementation with vitamin D has no effect on dynamic muscle strength in older women as long as there is no deficit [29].

In older men, it has been observed that sodium bicarbonate supplementation is not effective, and therefore no favorable effects are obtained in measures of muscular performance [31]. In addition, coenzyme Q10 also appears to be ineffective at reducing fatigue and improving aerobic and anaerobic function in older men [34].

However, the type of supplement or meal plan used, the dose, and the form of training or physical activity guidelines should be properly studied and adapted to each person individually, according to his or her nutritional needs, physical abilities, and level of health to prevent disease, improve general health, and increase performance, in some cases.

It was observed that, in most of the studies analyzed in this review, the subjects did not present significant changes in body composition. Three of the studies [27,31,34] did not evaluate body composition, so it was not possible to observe if there was any change after the interventions.

Beneficial changes in body composition have been found only in two studies conducted in women [32,34]. In the study by Sillampaa et al. (2010) [30], a decrease in BMI was observed, and therefore of body mass, and an increase in muscle mass in the legs after periodic two-cycle training, increasing intensity and volume in a cycle ergometer. In the other study [33], only body mass and fat-free mass were assessed, with an increase with differences in the group supplemented with HMB, arginine, and lysine compared to the placebo group. The rest of the studies [30,31,36] examined body composition but found no significant differences after the intervention period for any group.

Therefore, the only changes in body composition have been found in women. There is not enough evidence to establish a relationship between endurance training with nutritional strategies and men’s body composition. The most significant changes in the study by Sillampaa et al. (2010) [30] in women, in addition to the supplementation intervention, can be associated with the performance of specific endurance training, such as a programmed circuit or cycle periods in a cycle ergometer.

The main limitation of the present study is the shortage of studies evaluating the interactive effects of diet and endurance exercise in the elderly and those differentiated between older men and women in their results. In addition, the age of the participants in all the studies was not homogeneous. The endurance interventions in the studies were very different and the workload was not equally quantified. However, a strength of this study is that the conclusions for older people are safe because many comparisons have been made between different nutritional strategies that positively affect their health when doing endurance training.

Future research is required to optimize the health of the elderly and recommend endurance training and appropriate nutritional strategies. Future research should include intervention-based studies to see what types of endurance training are the most effective for older people and to improve their physical qualities. In addition, studies should consider long-term nutritional strategies to see if their quality of life improves. There is an obvious need for more research to assess the influence of endurance exercise and dietary intake of older people and to use performance indices as primary outcomes.

## 5. Conclusions

This review provides a compilation of nutritional strategies studied in older people undergoing endurance training. There are different nutritional supplements in both sexes that have positive effects, such as caffeine or high protein supplement with beta-alanine. In older women, supplementation with bicarbonate, HMB, lysine, and arginine also have shown positive effects on exercise performance. Health professionals should be aware of these strategies and consider their use for different interventions or supplementation protocols. No additional or exclusive effects were found in the population of older men. These high-quality nutritional practices provide a strong foundation for minimizing the adverse physiological effects of ageing on older people and maintaining health and wellbeing.

## Figures and Tables

**Table 1:** Evaluation of methodological quality (PEDro scale).

Lead Author, Year	1. Selection Criteria	2. Random Assignment	3. Hidden Assignment	4. Similar Groups	5. Blinded Subjects	6. Blinded Therapists	7. Blinded Evaluators	8. Adequate Follow-up	9. Intention to Treat	10. Comparison between Groups	11. Punctual Measures of Variability	Total Score
Ducan et al., 2014 [27]	Yes	Yes	Yes	Yes	Yes	No	No	Yes	Yes	Yes	Yes	8
McCormark et al., 2013 [28]	Yes	Yes	Yes	Yes	Yes	Yes	No	No	No	Yes	Yes	7
Verschueren et al., 2010 [29]	Yes	Yes	No	Yes	No	No	Yes	Yes	Yes	Yes	Yes	7
Sillanpää et al., 2010 [30]	Yes	Yes	No	Yes	No	No	No	Yes	Yes	Yes	Yes	6
Dawson-Hughes et al., 2010 [31]	Yes	Yes	Yes	Yes	Yes	No	Yes	Yes	Yes	Yes	Yes	9
Norager et al. 2005 [32]	Yes	Yes	Yes	Yes	Yes	Yes	no	No	Yes	Yes	Yes	8
Flakoll et al., 2004 [33]	Yes	Yes	Yes	Yes	Yes	Yes	Yes	No	Yes	Yes	Yes	9
Laaksonen et al., 1995 [34]	Yes	Yes	Yes	Yes	Yes	Yes	No	Yes	Yes	Yes	Yes	9

**Table 2:** Article reviews about physical exercise of endurance, nutritional supplementation, and diet; in chronological order following the published year.

Author and Year	Characteristics	Period	Intervention	Measures	Results	Conclusion	Type of Exercise	Corporal Composition
Duncan et al., 2014 [27]	*N* = 19 Sex: M = 9; F = 10 Age: 66 ± 2 years Non usual caffeine consumers	The sup. is given 60 min before the measurements	G 1: Caffeine (3 mg of caffeine x kg^−1^ body mass) G 2: Placebo (3 mg dextrose x kg^−1^ body mass)	Test fitness The Rikli and Jones Senior fitnessManual Dexterity Turning Test	G1 significantly improved performance compared to G2. In all analyses, gender was not significant	Acute caffeine intake improves functional performance and manual dexterity in older people.	Non training. The participants were physically active.	There were no pre- or post-intervention data regarding BC.
McCormack et al., 2013 [28]	*N* = 44 Age: 70.7 ± 6.2 years G1: *N* = 16 / Sex: M = 11; F = 5 G2: *N* = 15 / Sex: M = 5; F = 10 G3: *N* = 13 / Sex: M = 6; F = 7	12 weeks	G1: twice a day, Ensure high protein (ONS) G2: twice a day, ONS plus 800 mg beta-alanine G3: twice a day, ONS plus 1200 mg beta-alanine	Submaximal discontinuous test in cyclo-ergometer Manual grip dynamometer 30-sec sit-to stand (STS)DEXA	G2 and G3 show significant improvements in their PWCFT and were not significantly different. No improvements in GRIP G3: improvement in 30s STS test There were no significant changes in BC for any G.	ONS strengthened with beta-alanine can improve physical work capacity, muscle quality, and its function in older men and women.	No training	There were no differences in body mass, fat free mass, or fat mass.
Verschuerenet al., 2011 [29]	N: 113 Sex: F Age range: 70–80 years G1: *N* = 28/Age: 79.8 ± 5.3 G2: *N* = 26/Age: 80.3 ± 5.3 G3: *N* = 28/Age: 79.6 ± 5.2 G4: *N* = 29/Age: 78.7 ± 5.6	6 months	G1: VPT 3 times / week + 880 IU Vit D G2: VPT 3 times / week + 1600 IU Vit D G3 (control): 880 IU Vit D G4 (control): 1600 IU vitamin D	Muscle strength: knee extension with dynamometer CT scan of the thigh	The VPT program did not increase muscle strength, the FM, hip BMD, or serum Vit. D levels compared to a program without exercises. Ingestion of 1600 IU of Vit. D produced a greater increase in serum Vit D levels compared to a dose of 880 IU, but there were no differences in muscle strength between both G.	The strength and MM. do not change significantly The VPT does not offer additional improvements than that provided by vitamin D. A higher dose of vitamin D does not demonstrate muscle benefits in dynamic muscle strength, hip BMD, or serum vitamin D levels compared to conventional doses.	Static and dynamic exercises on a vibrating platform 3 times per week.	No significant differences in FM. G1: BMI: 27.5 (SD 2.7), FM, (cm3): 69.6 (SD 11.3) G2: BMI: 26.4 (SD 4.4), FM. (cm3): 67.3 (SD 8.0) G3: BMI: 27.4 (SD 3.7), FM. (Cm3): 72.1 ± 10.2
Sillanpää et al., 2010 [30]	G1: *N* = 21/Age: 53 ± 8/Sex: F G2: (control) *N* = 9/Age: 53 ± 8/Sex: F	21 weeks	G1: endurance training (cycle ergometer) 2x week + FNR: 47 ± 6% CH, 19 ± 3% protein, 32 ± 4% fat. G2 (control): No training + FNR	Maximum pedaling force Knee Extender Force DEXA	No improvements in knee extender strength. There is an increase in blood cortisol (32.7 ± 51.3%)	Endurance training with a bicycle twice a week increases the maximum pedaling power, but not muscle strength.	Cycle ergometer: Periodic training in two cycles: Increasing intensity and volume (from 30 min aerobic to 90 min)	G1: significant decrease in BMI and increase in MM in legs. Initial data: G1: Body Mass: 66 ± 9 kg/BMI: 25.1 ± 2.6/% fat 37.4 ± 5.1 G2: Body Mass: 66 ± 8 kg/BMI: 23.4 ± 2.0/% fat: 32.1 ± 6.1
Dawson-Hughes et al., 2010 [31]	Sex: F G3 (control): *N* = 49/ Age: 62.7 ± 7.4 G4: *N* = 42/Age: 61.7 ± 7.8	3 months	G1: (control) microcrystalline cellulose capsules G 2: (treatment) 67.5 mmol/day of sodium bicarbonate (sodium, potassium or sodium chloride) in gelatin capsules. Everyone took a calcium triphosphate sup. and a multivitamin with Vit. D3 daily with breakfast	1-RM/Leg press and knee extension Manual dynamometry Cybex II isokinetic dynamometer Blood and urine tests	Sodium bicarbonate sup. was well tolerated and the NAE decreased. NAE was inversely correlated with the change in performance measures. Sodium bicarbonate increased leg press power to 70% of an 1-RM and improved other performance measures.	Sodium bicarbonate ingestion decreased nitrogen excretion and sodium bicarbonate-induced decrease in NAE was associated with a reduction in nitrogen excretion. Sodium bicarbonate can reduce age-related loss of muscle performance and mass in older women.	No Training	There were no data regarding BC
	Sex: M G1 (control): *N* = 35/Age: 64.2 ± 8.2 G2: *N* = 36/Age: 63.8 ± 8.3				Sodium bicarbonate sup. was well tolerated and decreased excretion of NAE. The NAE was not correlated with any of the performance measures. The sodium bicarbonate treatment did not have a significant effect on muscle performance in men.	Sodium bicarbonate sup. was well tolerated but had no favorable effects on selected measures of muscle performance in men. The reason may be due to the dose in relation to body size.		
Norager et al., 2005 [32]	*N* = 30 Age: 74.7 ± 5.5 *N* = 15/Sex: F *N* = 15/Sex: M		G1: caffeine 1h before exercise (6 mg/kg) G2 (control): placebo G1 and G2: No caffeine 48 h before. High carb diet 1 day before.	Maximum voluntary isometric force of arm flexion Cycloergometer test Walking speed: 15 m. Perceived effort	G1: improves submaximal isometric strength by 54% and reduces the perceived effort in 5 min by pedaling by 11%. G1 had no significant effect on reaction times or movements	Caffeine sup. increases cycling endurance, isometric flexural strength of the arm, and perceived exertion during cycling in older people.	There was no training	There were no differences. Initial data: Height: 164.3 ± 9.2 m Body Mass: 72.1 ± 13.4 kg
Flakoll et al., 2004 [33]	Study 1: G1: *N* = 13/Age: 84.2 ± 1.6/Sex: F G2: (control) *N* = 10/Age: 81.1 ± 1.8/Sex: F Study 2: G1: *N* = 14/Age: 71.5 ± 1/Sex: F G2: (control) *N* = 13/Age: 71.5 ± 1.5/Sex: F	12 weeks	Study 1 and 2: G1: Orange flavor drink sup. (calcium HMB; arginine; lysine hydrochloride, and ascorbic Ac) Study 1: G2 (control): Drink (maltodextrin and ascorbic acid) Study 2: G2 (control): Drink (nitrogen + non-essential amino acids and ascorbic Ac)	“Get-up-and-go” functionality test Knee extender and knee flexor force Grip Force: Handgrip	G1 obtained a 17% improvement in the “get-up-and-go” test. G1 increased the circumference, leg strength, and grip strength	Sup.with HMB, arginine, and lysine improve functionality, strength, FFM, and protein synthesis, suggesting that this nutritional strategy affects muscle health in older women.	No training	G1: increases FFM (0.7 ± 0.3 kg) compared to subjects with a placebo sup. (0.0–0.3 kg)
Laaksonen et al., 1995 [34]	*N* = 19 Elderly men: *N* = 8/Age: 60–74 y/Sex: M Young men: *N* = 11/Age: 22–38/Sex: M	6 weeks separated by a 4 week rest period	G1: Ubiquinone + Placebo G2: Placebo + Ubiquinone Treatment phase: 120 mg ubiquinone + fish oil. Placebo phase: 120 mg of placebo + fish oil.	Ergometer endurance exercise tests Muscle biopsy to determine ubiquinone.	The concentration of ubiquinone in blood increased after sup. in older and younger people. The time to fatigue was longer after placebo intake than after the treatment phase.	Ubiquinone was ineffective in reducing fatigue and improving aerobic and anaerobic function and was also ineffective as an ergogenic aid in trained young and older men.	There was no training of the study, but they were trained subjects.	There were no data regarding BC.

Anthropometric (ANTP); body composition (BC); bone mineral density (BMD); body mass index (BMI); carbohydrates (CH); computerized tomography (CT); dual-energy x-ray absorptiometry (DEXA); everyday activity (EDA); energy expenditure (EE); feminine (F); Finnish nutritional recommendations (FNR); fat mass (FM); fat free mass (FFM); group (G); hand grip dynamometer (GRIP); beta-hydroxy methyl butyrate (HMB); international units (IU); masculine (M); muscular ass (MM); repetition maximum (RM); net acid excretion (NAE); oral nutritional supplement (ONS); physical work capacity at the fatigue threshold (PWCFT); repetition maximum (RM); supplement (Sup); sit-to-stand (STS); Vitamin D (Vit. D); Vitamin D3 (Vit. D3); vibrating platform training (VPT).
